# Promotion of Elementary School Students’ Health Literacy

**DOI:** 10.3390/ijerph17249560

**Published:** 2020-12-21

**Authors:** Elke Knisel, Helge Rupprich, Annika Wunram, Markus Bremer, Christiane Desaive

**Affiliations:** 1 Department of Sports Science, Faculty of Humanities, Otto-von-Guericke-Universität Magdeburg, 39106 Magdeburg, Germany; a.wunram@hotmail.de (A.W.); markus.bremer@st.ovgu.de (M.B.); christiane.mcconelldesaive@ovgu.de (C.D.); 2 Faculty of Social Sciences, Media, and Sports, Institute of Sport Science, Johannes Gutenberg University Mainz, 55128 Mainz, Germany; helge.rupprich@uni-mainz.de

**Keywords:** health literacy, physical activity, children, elementary school

## Abstract

Health literacy is an important outcome of the discussion of school-related health education and health promotion in the 21st century. Although the improvement of health literacy at an early age is increasingly recognized and few interventions show the development of children´s health literacy, still there is little research in this area. The purpose of the study was to examine the enhancement of health literacy among children in a physical activity-based program at elementary school. In total, 137 students aged 6–12 years participated in the program, which included health knowledge transfer in child-appropriate games and exercises. Participants´ health literacy was assessed using the HLS-Child-Q15-DE at the beginning and the end of the program. The instrument measures the access, understanding, appraisal and application of health-related information on a four-point Likert-type scale. As expected, the Wilcoxon signed-rank test revealed significant increases in self-reported health literacy over time. The results show that the degree of change in health literacy was not associated with gender or age. The results suggest that the physical activity-based program has the potential to improve elementary school children´s health literacy, even though in a single group pilot study.

## 1. Introduction

Health literacy is an important outcome of the discussion of school-related health education and health promotion in the 21st century [1]. The acquirement of skills which determine the individual motivation and ability to gain access to, understand and use health information [2] in childhood seems to be vital. Health literacy is a multidimensional approach with various definitions [3,4,5,6,7,8,9]. Common to these definitions is that individual skills to obtain, process and comprehend health information and services are needed to make suitable health decision [10]. A broader view of health literacy encompasses not only the individual perspective but also public health literacy [10,11,12,13]. This paper uses the definition by (Sørensen et al. (2012), p. 3) who states that health literacy “is linked to literacy and entails people’s knowledge, motivation and competences to access, understand, appraise, and apply health information in order to make judgments and take decisions in everyday life concerning healthcare, disease prevention and health promotion to maintain or improve quality of life during the life course” (Sørensen et al., 2012, p. 3; Sørensen et al., 2013, p. 2) [14,15]. In their review Sørensen et al. (2012) clustered different perspectives and described the three domains of health “healthcare, disease prevention and health promotion” with “being ill, being at risk and staying healthy” [14,15]. Therefore, this definition integrates both the individual and public health perspective on health literacy, and explains the knowledge and competencies required to meet the demands of individual life and modern society [2,16].

Despite this understanding of health literacy, there is limited consensus in health education research regarding the knowledge and skills elementary school children should possess for making appropriate health promoting decisions and actions [6] and therefore to be a “health literate child” [13,17] describe health literacy as an individual competency which comprises theoretical and practical health knowledge, critical thinking, self-awareness, and citizenship, and corresponding learning conditions to develop students’ health literacy at school. 

So far, there are only a few studies that have examined the promotion of health literacy in connection with physical activity. Based on the three-level concept of health literacy of Nutbeam (2000) [18], the program IMOVE by Bruselius-Jensena, Høstgaard Bonde and Hellesøe Christensen (2016) [19], for example, should help Danish primary school students to develop health literacy related to physical activity. Physical activity can also be found as a component of intervention measures, e.g., in the study by Aghazadeh and Aldoory (2020) [20]. However, there are hardly any physical activity programs yet found to improve health literacy. Following the idea that the promotion of health literacy could be more successful when practically adjusted to a specific topic, it seems promising using physical activity as a tool for teaching different health-related skills and knowledge. The approach of “learning by playing” could be especially essential and effective with young kids. In various literature reviews, a number of approaches were identified and studies performed for youth or secondary school students [6,8,21,22] Paakkari et al. (2020) [22], for example, compared the level of health literacy of 15-year-old students in 10 European countries (N = 14,590) using the Health Literacy for School-Aged Children (HLSAC) as an instrument. The findings revealed that 13% of the students reported low health literacy, whereas 67% had a moderate level of health literacy, and 20% reached a high level. Compared to the other countries, the mean score in Germany (N = 1429) was low, 16% of the German students showed a low level of health literacy. The rate of moderate and high health literacy was 71.2% and 12.8%, respectively. Nearly the same proportion of low health literacy was exposed in a representative study by Sukys et al. (2019) [23] among Lithuanian students from the 7th to 10th grades. The highest mean scores in the study of Paakkari et al. (2020) were found in Macedonia and Finland [22]. The Finish result corresponds to the study of Paakkari et al. (2017) [24] with seventh and ninth graders (N = 3833) who demonstrated that 33% of the participants showed a high level of health literacy and 60% had a moderate level of health literacy. 

In line with the international discussion, similar results are lacking for children under the age of ten. This may explain the scarcity of measurement tools of health literacy for children aged 6–12 years, found in the reviews of Guo et al. (2018), Okan et al. (2018) Ormshaw, Paakkari and Kannas (2013), and Perry (2014) [7,25,26,27]. According to Bollweg and Okan (2019) [3], the concepts behind the existing measurement tools differ and consequently the health literacy (HL) components and the health areas they address: “Accordingly, there is a need for comparable and validated tools designed to assess children’s HL”.

One of the few validated measurements is the screening tool Newes Vital Sign (NVS) [28]. A newly measurement approach for German-speaking children aged 9 and 10 years is the HLS-Child-Q15DE, which is an age-adapted version of the HLS-EU-Q to children [29,30]. Due to the lack of measurement instruments, there are still less intervention studies in the age group 6 to 13 years. Most of the interventions presented took place within a school setting with a medical background (Guo, 2018, Okan, 2015; Perry, 2014) and relate either to teacher training or programs for students [7,9,27].

A 6-year health literacy curriculum, called “Wellness”, targeting US low-income youths from third grade to eighth grade was designed by Diamond, Saintonge, August and Azrack (2011) [31]. The experiential learning approach with games and activities refers to an understanding of body function and food intake and making healthy choices, including exercise tips. The preliminary findings revealed an increase in knowledge and improved healthy behaviors. A Saturday school program in the United States by Robinson, Calmes and Bazargan (2008) [32], combining a reading skills and asthma-education program, had a positive impact on asthma-related outcomes with children aged 6–14 years with asthma. Franze et al. (2011) [33] developed a teacher-training program called GeKo^KidS^ in Germany. The target group for the ex-curricula teaching of health information were 5th grade students aged 9–13 years. The evaluation included medical examinations and self-completion questionnaires for the students. The student questionnaire consisted of self-developed short scales for the measurement of different domains of health literacy, such as knowledge, communication, attitude, self-efficacy, and health behaviors [34]. The authors found associations of the health literacy domains, health behaviors and subjective health. The scales on attitude, communication and self-efficacy were positively linked to each other, as well as to health behaviors and subjective health [34]. The authors reported a high level of acceptance and the appropriateness of the intervention program [33]. The aim of the program, Read Wyoming by Benham-Deal, Jenkins, Deal and Byra (2010) [35] was to examine the impact of a nine-month teacher training on elementary school teachers and students. Significant increase in elementary teachers’ confidence to teach health education when infused with literacy instruction and the practice of integrating reading and health instruction were observed. The use of HEAP (Health Education Assessment Project) assessment by the teachers shows a moderate effect on students´ knowledge about injury prevention and safety, and health skills of accessing valid sources of health information and services. Aghazadeh and Aldoory (2020) [20] implemented a health literacy intervention program for second and third grade students in two US elementary schools with different socioeconomic levels. The program was designed to improve health knowledge, self-efficacy, health decision-making, listening, and oral communication. The intervention programs comprised topics as physical activity, nutrition, safety, and communication with health care providers. Materials, like books, vocabulary games, and activities with nutrition labels, were used by the teachers and health experts in different lessons. The findings show a significant enhancement in health literacy measured with the NVS in the low-income school in comparison to the second school. No impact of the intervention was found in perceived health status, self-efficacy or communication confidence. The authors infer the value of school-based interventions in elementary school to improve appropriate health literacy skills.

To sum up, although the improvement in health literacy at an early age is increasingly recognized and few interventions show the development of health literacy, there are still less studies in the age group 6 to 13 years [7,9]. Furthermore, it is difficult to compare the reported intervention studies, as the programs differ significantly from one another and a detailed overview of their content regarding the theoretical background and their effects are not always demonstrated. Okan (2019) [36] emphasized for future interventions to describe concrete steps for the process of accessing, understanding, appraising and applying health-related information to examine effects more closely. Consequently, our purpose was improving primary school children´s health literacy by measures related to this process in a physical activity-based program in school holidays.

## 2. Materials and Methods 

### 2.1. Study Design and Participants

137 elementary school children aged 6–12 years (mean age 8.22 ± 1.34) participated in four health-related physical activity programs five days each. The participants were recruited from the holiday camps of five elementary schools in the city of Magdeburg, Germany. The holiday program was free to all children. 

All children in the recruited camps were invited to participate and the parents of 500 students agreed to participate in the study. In total, 363 students were excluded from the analysis due to an incomplete questionnaire, fluctuation within the program, lack of presence at the day of data collection or illness. Therefore, the final sample consisted of 57 girls (mean age 8.11 ± 1.28 years) and 80 boys (mean age 8.3 ± 1.38 years). The children´s ages were dichotomized as “first and second graders” aged 6–8 years old (n = 83; 35 girls and 48 boys) and as “third and fourth graders” aged 9–12 years old (n = 54; 22 girls and 32 boys) according to the age groups in German elementary school classes.

A pretest/post-test study was conducted. Data were collected on the first and the last day of each program, respectively. After an introduction into the program and an explanation of the purpose of the study, the students were asked to fill out the questionnaire at the beginning of each camp. Each student participated voluntarily in the program and the data collection. Anonymity of answers was guaranteed. 

### 2.2. Physical Activity Program

The focus of the program was the integration of health literacy into a physical activity program. Therefore, we worked out a health literacy curriculum with child-appropriate games and exercises linked with physical activity. Each of the four health-related physical activity programs in school holidays continued for five six-hour days. They took place in the school holidays at Easter (one program), summer (two programs) and autumn (one program) in 2019.

The health literacy curriculum was developed to promote health competencies according to the integrated conceptual model of health literacy of the European HLS-EU consortium [14]. It is based on four topics, namely to promote ability (1) to seek and find health-relevant information, (2) to comprehend health-relevant information, (3) to interpret and evaluate health-relevant information and (4) to communicate and use the information to improve health [14]. According to L. Paakkari and O. Paakkari (2012) [13], who emphasized different core elements to define health literacy as a learning outcome, we introduced theoretical knowledge and practical information, critical thinking, and self-awareness in our exercises. 

To perform the theoretical approaches of the health literacy curriculum, the instructors, for example, carried out exercises to improve body perception and body awareness, played perceptual games, and conducted discussions with their groups that reinforced the topics. For example, after the accomplishment of 30 jumping jacks, the children measured their pulse rate. The questions of what happens in the body when moving and the subsequent reflection was intended to give the children an understanding of the benefits of physical activity. While exercising, they perceived higher pulse rates, sweating, changes in body temperature and skin color, as well as faster breathing, and they learned to interpret these changes. In another so-called energy game, for instance, each child was asked to collect playing cards with various foods as fruits, vegetables, sweets and drinks depicted upon them and assign the cards with the foods shown to the categories "health-promoting foods" or "non-health-promoting foods". After the children found out how the foods differ, what amounts you can eat, where they can be found in the supermarket etc., a relay game was played. The playing cards should be collected from each group as quickly as possible and assigned correctly. Based on the idea of learning critical thinking, the participants learned to analyze health information critically [18,37]. In role-playing games, for example, a boy who is reluctant to walk to school is convinced by his mother that it is better to walk to school than being driven by car. The children then walked a certain distance and other children were pulled with the cart. After the role play the pros of physical activity were discussed with the children.

Each day ended with relaxation exercises like “bear massage” with music, dream trips with birds, and a reflection about when, why and what effects relaxation has. The instructors also fostered the children to deal with the health topics with their families at home. For example, the children were asked to bring foods from the two food categories the other day or to compare the pulse rate of their parents or sisters with their own after moving. 

The instructors were current university students of sport science with a trainer qualification. Before the program began, all instructors and the educators of the participating elementary schools attended one day of training at the university. This training focused on program logistics and knowledge transfer how the instructors could implement the health literacy curriculum into the physical activity program with the support of the educators.

### 2.3. Measures

Participants´ health literacy was assessed using the HLS-Child-Q15-DE [3,25,29,30,38,39] at the beginning and the end of the physical activity programs. The instrument is a German adaptation of the European Health Literacy Survey Questionnaire [15]. The tool assesses the subjective health literacy of 9 to 10-year-old children with respect to the health domains of healthcare, disease prevention and health promotion. The 15 items measure the perceived difficulty of accessing, understanding, appraising and applying health-related information (e.g., “How easy or difficult is it for you to find out how you can relax the best”) on a four-point scale ranging from 1 (very difficult), 2 (fairly difficult), 3 (fairly easy) to 4 (very easy). The authors added “don’t know” as an additional response category [25,30]. Lower scores refer to perceived difficulties in dealing with health information. The Cronbach’s alpha (α = 0.79) indicated good internal consistency [25,30]. According to the original HLS-EU-Q, a mean score is calculated for children with a maximum of 20% missing responses which correspond to valid responses to at least 12 of the 15 items [25,30].

### 2.4. Ethics

All research procedures were conducted with strict adherence to ethical principles as set forth by the universities involved, and ethical approval was obtained from the Ethics Committees. All children took part in the study voluntary and could discontinue their participation at any time. Prior to study, all parents provided written informed assent. 

### 2.5. Statistical Analysis

A normal distribution was observed using the Kolmogorov–Smirnov test to assess the normality of the data (α > 0.05). The Wilcoxon signed-rank test for dependent samples was applied to study the participants’ change in health literacy from pretest to posttest. This frequently used nonparametric test procedure assesses, whether the central tendencies of two dependent samples are different. The null hypothesis is that the differences have a distribution centered at about zero. The absolute differences were ranked and the positive and negative rank positions added up [40]. The criterion for statistical significance was *p* < 0.05. To measure the strength of the treatment response effect sizes were computed using Pearson correlation coefficient r. Values can be interpreted as follows: r = 0.10 small effect, r = 0.25 = medium effect, r = 0.40 = strong effect [41]. 

To assess age- and gender-specific differences in the change in health literacy, the one-way analysis of variance with repeated measurements was applied. Homogeneity of variances could be assumed after using Levine’s test which showed equal variances (*p* > 0.05). The partial eta squared reported the effect size and is converted according to Cohen (1988) with f = 0.10 small effect, f = 0.25 = medium effect, f = 0.40 = strong effect [42].

To compare the results on the general health literacy index (HL_index = HL_mean – 1) * (50/3), the data were transformed as in the European health literacy survey (HLS-EU) study to a unified metric from 0 to 50 with the four levels “inadequate” (0–25), “problematic” (>25–33), “sufficient” (>33–42) and “excellent” (>42–50) health literacy [43]. SPSS Statistics for Windows (IBM Corp, Armonk, NY, USA) version 25 was used for the data analyses.

## 3. Results

As expected, the Wilcoxon signed-rank test revealed significant increases in self-reported health literacy over time (see Table 1). The value of the effect size of Pearson correlation coefficient is r = 0.42 which represent a strong correlation [41]. Girls and boys (see Table 1) significantly improved their ability to access, understand, appraise and apply health-related information into the contexts of health care, disease prevention, and health promotion. The Pearson correlation coefficients are considered a strong correlation. The one-way analysis of variance with repeated measurements revealed that the degree of change in health literacy was not associated with gender (F (1, 135) = 0.143, *p* = 0.585, η^2^ = 0.002, f = 0.05). 

The findings show that the first and second graders and the third and fourth graders (see Table 1) improved their health literacy significantly over time. The value of correlation coefficients represents a strong correlation. However, the data show significant age-specific differences at the beginning of the program (z = −3.1, *p* = 0.002, r = 0.3, n = 137). The results of the ANOVA show no age-specific effects of the program (F (1, 135) = 0.083, *p* = 0.773, η^2^ = 0.001, f = 0.05, n = 137).

The Wilcoxon signed-rank test show a significant increase in the three health domains of healthcare disease prevention and health promotion (see Table 2). The correlation coefficients are interpreted as moderate correlations. However, a significant positive change was observed in only three of the four health dimensions with a strong effect in the dimension of accessing health information and medium effects in understanding and applying health-related knowledge (see Table 3). 

**Figure 1:**
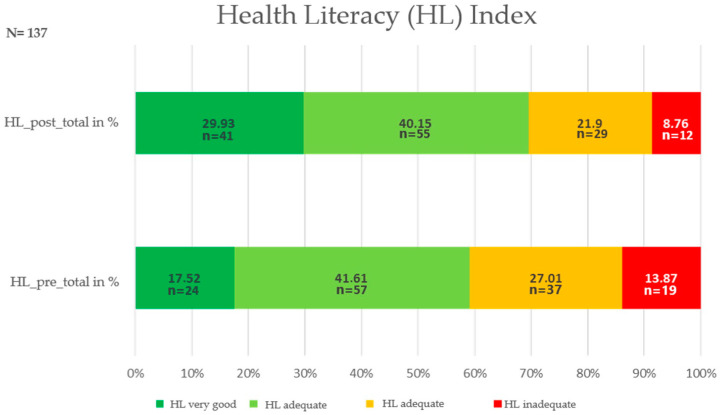
Health literacy index.

The health literacy index increased significantly from the pre-testing (M_pre_ = 34.06, SD = 7.9) to the post-testing (M_post_ = 36.93 (SD = 8.2) with a strong effect (z = −4.93, *p* < 0.001, r = 0.42). The data show a decrease in inadequate, problematic, and sufficient health literacy of 5.11% and 1.46%, respectively, whereas the level of excellent health literacy increased by 12.41% (see Figure 1).

## 4. Discussion

Our results show that the degree of health literacy is, on average, quite high. The participants reported that the dealing with health information is fairly easy or very easy. To our knowledge, our study is the second one applying the HLS-Child-Q15-DE. The first study was performed by Bollweg et al. (2020) [30] and Fretian et al. (2020) [29] with 907 German students aged 8–12 years in North Rhine-Westphalia. Bollweg et al. (2020) [30] tested the psychometric properties of the instrument and Fretian et al. (2020) [29] explored the subjective health literacy associated with factors such as self-efficacy or motivation. In line with our results, the children´s health literacy was rather high (M = 3.34; SD = 0.37) [29,30]. Corresponding to our results, Brown et al. (2007) [44] found in their study in the US that most of the students (N = 1178) aged 9–13 years reported the understanding of health-related information to be easy. 

Our findings suggest that our physical activity-based program has the potential to improve elementary school children´s health literacy. The level of self-reported health literacy and the health literacy index increased. The program’s overall effect size was strong. However, it must be recognized that the sample size of the study is rather small and the data collected for the current study are from a single group pilot study that did not include a control group. The program offered exercises and games to develop skills to obtain, process and comprehend health information. Results from this study are consistent with research, which has demonstrated the benefits for elementary school children arising from health literacy interventions inside and out of the classroom. Children´s education is the focus of school and health literacy approaches have to be integrated and adapted to age-specific educational objectives [45,46]. These health literacy approaches demand more a promotion-focused model and a resource-based health perspective, such as the salutogenic paradigm by Antonovsky (1996) [47], rather than a deficit perspective in structuring the curricula and the acquisition of skills desired of health literacy interventions in elementary school [6,21,48,49]. Correspondingly, the WHO (2013, 2017) suggested introducing health literacy approaches into the health-promoting school framework. 

According to the salutogenic paradigm, exterior resources, such as social structures and peer group support, can benefit children´s acquirement of health literacy-related knowledge and skills. Social formal support structures in different classes and cross-subject teaching, as well as informal support structures in ex-curricula teaching, such as a holiday program at elementary school, can help children to access health information and accomplish health-literate-related tasks or actions. However, there is a need for educational research into what kinds of learning situations in which social structures promote health literacy effectively as elementary school age may be considered an important life stage for the development of health resources and health-related competencies and behaviors [6]. Therefore, intervention studies should further consider the social structures of school and after-school programs and the different school subjects (e.g., Biology or Physical Education) in which the teaching and performing of health literacy takes place [6]. This type of research may help to design programs tailoring health-information to different learning situations.

Our health literacy curriculum was based on the four health dimensions of finding, understanding, appraising and applying health information. The results suggest that the sub-process of assessing health information is more difficult than finding, understanding, and applying health information. To interpret and evaluate health-relevant information to make decisions requires critical thinking and critical health knowledge transfer, which is seen as the core dimension of health literacy [13,18]. In our program, the instructors supported the finding and understanding of health information systematically, with various games and exercises and the applying process with “homework”. However, the program did not have the potential to develop the process of appraising health-relevant information about physical activity or healthy food. The study IMOVE by Bruselius-Jensen et al. (2016) [19] showed similar results. The authors found that most of the classroom dialogue occurred at the functional and interactive health literacy level, which means the ability of understanding the relationship of everyday practice and step numbers and to apply this knowledge in planning and practicing physical activity. Less classroom-based discussions were on the critical health literacy level and the influences of social determinants on physical activity levels. The sub-process of appraising health information affords some critical analysis by linking the knowledge to the children´s own attitudes and experiences [50] to make health decisions. The child´s competence to interpret and evaluate health information may depend, to a considerable extent, on the parents’ health literacy. Studies show an impact of parental health literacy on children’s health outcomes [51,52,53,54]. If we assume that parents’ health knowledge and health behavior have a significant effect on this sub-process, further intervention studies should target and assess parents´ health literacy [54] to meet the health literacy needs of children [52]. Digital tools with commented movement games and exercises for children and parents accompanied by health information could support the acquisition of health skills for the whole family—even in phases of lockdown, such as during the current pandemic. According to Sanders et al. (2009) [52], it is important to regard the connection of children´s and parents´ health literacy to evaluate intervention results. As stated by Bruselius-Jensen et al. (2016) [19], the ability to know and understand the impact of physical activity on the body and to critically deal with physical activity recommendations to draw conclusions for the own daily exercise seems to be an approach for further physical activity-promoting programs for children. 

Our findings imply that the acquisition of health-related knowledge and its application, associated with physical activity, indicates an approach to enhance children´s health literacy in a physical activity-based ex-curricula program. Our approach to introduce health knowledge with different materials and combine them with brief health information in different exercises is in accordance with the argument of DeWalt and Hink (2009) [55], that teaching health information closely related to behaviors and outcomes is more promising to improve health behavior. However, so far, only a few studies dealt with the teaching of health literacy in connection with PE classes or ex-curricula physical activity programs. Future research of long-term effects of these approaches on health behavior in general and health-related physical activity in particular is required. Empirical evidence regarding gender- or age-specific differences of children´s health literacy is rare [29]. A few studies investigated factors associated with health literacy, such as health knowledge, health-related attitudes or behavior concerning participant "age and gender" [34,45]. Paakkari et al. (2017) [24] showed that the level of health literacy was lower for boys in the seventh and ninth grade than for girls and Yu et al. (2012) [56] found that the elementary school boys´ health knowledge level was lower than that of the girls. Although age- or gender-specific differences in health literacy have been observed, this was not the case in our study. Girls and boys enhanced their level of health literacy. Recently, Fretian et al. (2020) [29] also found no differences in health literacy in German fourth graders with regard to gender and age by using the HLS-Child-Q15-DE. 

In the discussion about potential reasons for gender differences it is argued that the school environment may encourage girls more than boys. This position is consistent with the results of the PISA study in reading literacy [57,58]. One of the reasons that we did not find any differences between girls and boys regarding health literacy may be that the influence of the school’s social environment is less impactful than that of parents of eight-year-old children, and gender differences become apparent later. This gender-gap was observed by Klecker (2005) [59] in reading literacy, comparing fourth, eighth and twelfth graders. As grade-level increased, girls scored higher than boys in reading literacy [59]. Schmidt et al. (2010) [34] found that girls had more health knowledge and are more interested in health-related topics. This is especially true in the area of nutrition, whereas boys are more interested in sport and exercise. In our program, the health information focused on healthy food and physical activity, which improved both girls´ and boys´ health literacy. 

The findings of Driessnack et al. (2014) [60] revealed a positive correlation between the level of health literacy and age in children aged 7–12 years. Even if we dichotomized “first and second graders” aged 6–8 years old and “third and fourth graders” aged 9–12 years old, we found no differences between the two age groups regarding the enhancement of health literacy. However, the level of health literacy was higher in the second age group than in the first age group at the beginning of the physical activity program. Additionally, the average age was low in the first age group with 7.31 years and in the second age group with 9.61 years. Subsequently, as postulated by Fretian et al. (2020) [29], we need longitudinal studies with broader age ranges to research the development of health literacy through the different phases of childhood and adolescence. 

While this study offers contributions to future research in the area of children´s health literacy, the conducted study does have limitations. Firstly, our study has a quasi-experimental study design and no RCT and it is possible that selection bias has affected the results. Additionally, the presented effect sizes are based on correlations and do not represent credible causal estimates. Effect sizes are larger for correlational studies than for causal studies [61]. Moreover, we did not compare the group of completers with the non-completers at the beginning of the program. These results may give implications for future tailored interventions. Regarding the questionnaire HLS-Child-Q15-DE, the items of each dimension is not the same number. The subscale “appraise” is represented by just one question. If the children did not answer this question, the entire appraisal dimension was not answered. Furthermore, the data are based on children´s self-reports, and social desirability response bias may lead to inaccurate self-reports and erroneous study conclusions [62]. Lane and Aldoory (2019) [63] and Okan et al. (2018) [25] underline combining subjective and objective measurements and consequently mixed methods tools to study children’s health literacy. The comparison of our results in self-reported health literacy with, e.g., the change in daily steps measured with pedometer, would give more information on the effects in the area of physical activity. Furthermore, according to Bollweg et al. (2020) [30], this performance-based measure may show to what extent self-reported health literacy measures are able to predict health-related outcomes in different behavior areas.

## 5. Conclusions

The purpose of the study was to develop an age-specific physical activity program with games and exercises for elementary school children to improve health literacy. The results show that the health-literacy-related measures, with a focus on finding, understanding, appraising and applying health information within a physical activity-based program were effective, even though in a single group pilot study. The enhancement of health literacy was not dependent on age or gender. So far, only a few intervention studies were conducted in the area of children´s health literacy, which is due to the few existing age-specific instruments. The HLS-Child-Q15-DE as quite a new instrument offers the possibility to study the level of health literacy of German elementary school children. Our exploratory study with this new instrument is an approach for further studies to investigate the effectivity of health literacy-related interventions in or outside of school. However, a validation in different languages is needed to compare the results.

For the design of future health literacy-related programs in PE and other classes or in after-school programs, it is essential to clarify which competencies are to be promoted regarding the educational objectives and the social structures. Therefore, a distinction must be made between, e.g., exercise-oriented health literacy programs and thus an area-specific model of health literacy and health-related physical literacy programs. Recently, the approach of physical literacy is within the focus of some intervention studies. 

## Figures and Tables

**Table 1:** Age- and gender-specific results.

All	Girls	Boys	First/Second Graders	Third and Fourth Graders
M_pre_ = 3.04	M_pre_ = 3.01	M_pre_ = 3.06	M_pre_ = 2.93	M_pre_ = 3.21
SD = 0.48	SD = 0.43	SD = 0.51	SD = 0.48	SD = 0.41
M_post_ = 3.22	M_post_ = 3.21	M_post_ = 3.21	M_post_ = 3.1	M_post_ = 3.4
SD = 0.49	SD = 0.47	SD = 0.51	SD = 0.49	SD = 0.44
z = 4.929	z = −3.214	z = −3.734	z = −3.69	z = 3.26
*p* < 0.001	*p* < 0.001	*p* < 0.001	*p* < 0.001	*p* < 0.001
n = 137	n = 57	n = 80	n = 83	n = 54

**Notes.** Abbreviations: M_pre_ = mean health literacy of the pre-test; M_post_ = mean health literacy of the post-test SD = standard deviation; z = z-value of the Wilcoxon signed-rank test.

**Table 2:** Results of the three health domains of health care, disease prevention and health promotion.

Health Care	Disease Prevention	Health Promotion
M_pre_ = 2.95	M_pre_ = 3.04	M_pre_ = 3.12
SD = 0.57	SD = 0.69	SD = 0.48
M_post_ = 3.12	M_post_ = 3.26	M_post_ = 3.27
SD = 0.6	SD = 0.62	SD = 0.5
z = 3.45	z = 4.22	z = 3.71
*p* = 0.001	*p* < 0.001	*p* < 0.001
r = 0.32	r = 0.36	r = 0.32
n = 137	n = 137	n = 137

**Notes.** Abbreviations: M_pre_ = mean health literacy of the pre-test; M_post_ = mean health literacy of the post-test; SD = Standard deviation; z = z-value of the Wilcoxon signed-rank test; r = Pearson correlation coefficient.

**Table 3:** Results of the health dimensions of accessing, understanding, appraising and applying health information.

Access	Understanding	Appraising	Applying
M_pre_ = 2.99	M_pre_ = 3.0	M_pre_ = 2.9	M_pre_ = 3.22
SD = 0.52	SD = 0.64	SD = 0.82	SD = 0.58
M_post_ = 3.24	M_post_ = 3.14	M_post_ = 3.0	M_post_ = 3.37
SD = 0.56	SD = 0.54	SD = 0.86	SD = 0.59
z = 5.02	z = 2.769	z = 1.16	z = 2.809
*p* = 0.000	*p* = 0.006	*p* = 0.245	*p* = 0.005
r = 0.43	r = 0.24	r = 0.1	r = 0.24
n = 137	n = 137	n = 137	n = 137

**Notes.** Abbreviations: M_pre_ = mean health literacy of the pre-test; M_post_ = mean health literacy of the post-test; SD = Standard deviation; z = z-value of the Wilcoxon signed-rank test; r = Pearson correlation coefficient.
